# Placenta Prævia

**Published:** 1854-07

**Authors:** O. H. Taylor

**Affiliations:** Camden, N. J.


Placenta Prævia.—by O. H. Taylor, M. D., Camden, N. J.—In examining the recent medical report from Gloucester County, N. J., I observe that Dr. Sickler has presented, with some interesting remarks, two cases of presentation of the placenta, in each of which the placenta was *spontaneously delivered* before the birth of the child.

The question, whether the result so happily effected by nature in these instances, should ever be promoted by art, is one that has been canvassed of late, by obstetricians, with deep interest and anxiety. Nor is this surprising, when we consider the extreme urgency of the dangers threatening the mother from hemorrhage, and the vital importance of the function of the placenta, even during labor, if at all protracted.

From my own observation and reflection on several cases of attachment of the after-birth over the *os uteri*, coupled with severe hemorrhage, which have occurred in my practice within the last twenty-eight years, I am induced to coincide with Dr. Sickler in opinion, not only as to the propriety and safety, but the *cbsolute necessity*, in certain circumstances, of removing the placenta before the delivery of the child. That the established method of turning the infant in cases of severe hemorrhage, during parturition, may be impracticable, without great delay, when the os uteri is but slightly dilated, and presents rigid and unyielding edges, every experienced practitioner is aware. The strength of the mother may be fatally exhausted before the hand can be introduced. Even in the most favorable cases, the condition of the placenta during the delivery, with much of its surface still adherent, and preventing the contraction of the bleeding vessels beneath the detached portions, is incalculably more favorable for an arrest of hemorrhage, than if it were entirely detached; and the question of danger to the child, from the removal of its external lungs before its mouth reaches the atmosphere, is one of time only. If there be a possibility of a rapid delivery, we should not hastily allow this danger to prevent us from giving the greatest possible security to the patient.

*Three* cases of placenta implanted over the os uteri, occurred to me in the early part of my practice, and their history proves how fatal may be the results of uterine hemorrhage, even during the act of turning and delivering the child. In each of these cases, the operation of perforating the after-birth was performed in the presence and by the counsel of the late distinguished professor James, of the University of Pennsylvania; Dr. Charles D. Meigs, now Professor of Obstetrics in Jefferson Medical College, being also present in consultation in one of these cases. All these cases terminated fatally in a very short time after the delivery of the infants, which were still-born. Neither of the mothers survived more than three hours, and all died from the exhaustion caused by excessive hemorrhage.

That the operation of removing the placenta immediately, when found to present itself at the os uteri, is capable of being performed with safety to both mother and child, under favorable circumstances, I am fully convinced; but as Dr. Sickler very pertinently remarks, "the advisability and determination of the conditions in which it should be practiced, must be decided by an induction from a larger number of cases than have as yet been submitted to the profession."

My own experience leads me to the conclusion, that when the placenta is fairly implanted over the os uteri, and is firmly attached throughout its borders, it can be removed before delivery, with more safety both to the mother and child, than can be secured by perforating or elevating the edges, and bringing the child down by the feet.

In nearly all the cases that I have witnessed, expulsive and efficient pains have been brought on soon after the placenta has been completely detached; and these pains have continued so as to produce the prompt expulsion of the child whenever the presentation has been natural.

Even when the complication is coupled with a preternatural presentation, it does not appear to me that the immediate delivery of the placenta is necessarily contra-indicated. It would be folly to dwell upon the imminent danger to the child in such cases; and if the hemorrhage be permitted to continue unchecked during the protracted delivery by the feet (supposing this to be possible in the case) what will be the fate of the mother? Should we not give her the advantage of that arrest of hemorrhage which appears so generally to follow the entire detachment of the placenta, even before its positive expulsion? Certainly we should, at least whenever the presentation or the condition of the patient can be ascertained to be such, that very prompt delivery by the feet is impracticable; for then the death of the child is insured at all events; and as has been already hinted, it is precisely when such prompt delivery is most easily effected, that the danger to the child from the previous detachment is least, and the advantage to the parent I think, indisputably the greatest.

I throw out these few suggestions to the profession, in the hope of inducing the report of every fact which may tend to decide one of the most important practical questions which has been mooted by obstetrical practitioners for many years. Uterine hemorrhage has long been the terror of both patient and physician, and anything which tends in the least degree to lessen its dangers, is worthy of the most profound respect and serious consideration.

Permit me, then, to offer, in concluding this note, the abstract of a recent case, in which the effect upon uterine hemorrhage produced by the expulsion of the placenta before the delivery of the child, is happily illustrated.

On the 27th of April, 1853, I was requested to visit Mrs. B., on account of a profuse and unnatural flow of blood from the uterus. She considered herself as being eight months advanced in pregnancy. At the time of my first visit, she was not complaining of much pain, though the hemorrhage was very considerable in amount.

A digital examination proved that the os uteri was but slightly dilated. I directed pulv. acetat. plumbi et opii, but found it necessary, also, to apply the tampon, by means of which the bleeding was restrained, and I was enabled to leave my patient in an hour.

After the lapse of eight or ten days, the flooding recurred, and the same treatment was repeated, with a similar result.

On the 15th of May following, being the 19th day after the first attack, I was again summoned to the case, and found the patient laboring under a hemorrhage quite as profuse as at the time of the first visit, or even more so. By examination per vaginam, I found the os uteri dilated about two inches. The placenta was evidently implanted immediately over it. By pressing the index finger firmly towards the right ilium, I detached a portion of the adherent placenta, and was able distinctly to recognize the presenting portion of the fœtus, which proved to be the head.

Periodic pains of the regularly expulsive character, were now established, but each was accompanied with an excessive discharge of blood.

The imminency of the danger to the mother from hemorrhage, induced me to decide that nothing would be forfeited by following any plan of action calculated promptly to arrest it. I therefore proceeded to separate the attachments of the placenta to the uterus in the hope that possibly I might be able to thrust the after-birth back from the orifice, and thus enable the head to engage itself in the superior strait. On making the attempt to push the placenta beyond the presenting part of the head, however, I found myself opposed and thwarted by the descent of a large portion of the mass into the vagina. The result accorded with the experience adduced from the history of other and parallel cases; for two or three more pains completely expelled the placenta into the vagina, and the hemorrhage then instantly ceased. In five or six minutes more the child was born, and although reasonably a little languid at first, it soon began to cry, and has since been a healthy and promising child.—*The New Jersey Medical Reporter.*

